# Liver Resection With Inflow Occlusion

**DOI:** 10.1155/1990/21454

**Published:** 1990

**Authors:** R. C. N. Williamson

**Affiliations:** Department of Surgery Royal Postgraduate Medical School Hammersmith Hospital, DuCane Road London W12 0NN UK


tumour was more aggressive or more advanced at the time of diagnosis; the figures only demonstrate that, without resection, some patients can live for prolonged periods of time (60% of them had multiple hepatic metastases). On the other hand, if we separate out the two patients who, for unknown reasons, survived more than 7 ½ years, then the other 11 patients died between 4 and 30 months (average survival 12 months), which is not significantly different to non resected patients; this is more clearly seen if we consider that 7 of 11 (63.6%) patients died between 4 and 12 months after surgery (average, 7 months). Recurrences of disease were always located in the abdominal cavity, and in 50% of cases in the same anatomical region; this fact has led some authors to suggest intraoperative radiation therapy, combined with the extended resection. At present, the value of this form of treatment is unproven.

My opinion is that this is a laudable effort in the treatment of a disease which had very little chance of being cured in stages further than I or II. Also, the results for stage V are too optimistic, and contrary to most published results. Readers should evaluate these results very critically and compare them with their personal experience.

E. Moreno Gonzalez, MD
Head Digestive Surgery Department
Professor of Surgery
Instituto Nacional de la Salud
12 de Octubre
MADRID 28041
SPAIN

### LIVER RESECTION WITH INFLOW OCCLUSION

### ABSTRACT

*Delva, E., Camus, Y., Nordlinger, B., Honnoun, L., Parc, R., Deriaz, H., Lienhart, A. and Huguet, C. (1989) Vascular Occlusions for Liver Resections — Operative Management and Tolerance to Hepatic Ischemia: 142 Cases. Annals of Surgery,* **209**, 211–218.

**The intra- and early postoperative courses of 142 consecutive patients who underwent liver resections using vascular occlusions to reduce bleeding were reviewed. In 127 patients, the remnant liver parenchyma was normal, and 15 patients had liver cirrhosis. Eighty-five patients underwent major liver resections: right, extended right, or left lobectomies. Portal triad clamping (PTC) was used alone in 107 cases. Complete hepatic vascular exclusion (HVE) combining PTC and occlusion of the inferior vena cava below and above the liver was used for 35 major liver resections. These 35 patients had large or posterior liver tumors, and HVE was used to reduce**

**the risks of massive bleeding or air embolism caused by an accidental tear of the vena cava or a hepatic vein. Duration of normothermic liver ischemia was 32.3±1.2 minutes (mean ± SEM) and ranged from 8 to 90 minutes. Amount of blood transfusion was 5.5 ± 0.5 (mean ± SEM) units of packed red blood cells. There were eight operative deaths (5.6%). Overall, postoperative complications occurred in 46 patients (32%). The patients who experienced complications after surgery had received more blood transfusion than those with an uneventful postoperative course ($p < 0.001$). The length of postoperative hospital stay was also correlated with the amount of blood transfused during surgery ($p < 0.001$). On the other hand, there was no correlation between the durations of liver ischemia of up to 90 minutes and the lengths of postoperative hospital stay. The longest periods of ischemia were not associated with increased rates of postoperative complications, liver failures, or deaths. There was no difference in mortality or morbidity after major liver resections performed with the use of HVE as compared with major liver resections carried out with PTC alone, although the lesions were larger in the former group. It is concluded that the main priority during liver resections is to reduce operative bleeding. Vascular occlusions aim at achieving this goal and can be extended safely for up to 60 minutes.**

### PAPER DISCUSSION

KEY WORDS: Liver resection, inflow occlusion, liver ischaemia

Most surgeons embark upon a major liver resection by exposing the bifurcations of the hepatic artery and portal vein at the hilus, outside the liver substance. If a hemihepatectomy is planned, occlusion of the relevant vascular inflow may help to demonstrate the best line of liver transection, as the ischaemic half becomes dusky. Opinions differ on whether to ligate and divide the right (or left) hepatic artery and portal vein at the outset[1] or merely to clamp them initially before locating and dividing their branches within the liver parenchyma as the resection proceeds[2]. Although preliminary clamping alone has the theoretical advantage of avoiding injury in the case of vascular anomalies, either technique is appropriate in experienced hands. More contentious is the problem of dealing with the right hepatic vein, especially during extended right hepatectomy for a large and posteriorly placed tumor. Some surgeons would mobilise and sling the inferior vena cava above and below the liver before approaching the right hepatic vein (and ligating it if possible). Professors Parc, Huguet and their colleagues go further and actually cross-clamp the cava above and below the liver for up to 90 minutes in difficult cases.

Caval clamping combined with inflow control (the Pringle manoeuvre) produces hepatic vascular occlusion similar to the anhepatic phase of liver transplantation. As the authors state, occluding the portal vein alone is dangerous in dogs, but fortunately man has sufficient portasystemic collaterals to decompress the portal system, so that occlusion of the entire hepatic inflow has relatively minor haemodynamic effects. Total hepatic vascular exclusion (i.e. concomitant interruption of the cava above and below the liver) produces a 50 per cent fall in cardiac output and can be criticised on the grounds that it is unsafe and rarely necessary[1]. The present paper indicates that the technique is at least safe. Among 85 patients requiring inflow control for major hepatectomy, no less than 35 had hepatic vascular exclusion while 50 did not. There were 3 deaths in each group (9 versus 6 per cent: NS), and the complication rate was almost identical (43 versus 44 per cent). Thus the haemodynamic effects of vascular exclusion are generally well tolerated (granted a skilled anaesthetist), though one patient whose cava had to be clamped in a hurry to control unexpected bleeding developed circulatory embarrassment. As to the necessity of vascular exclusion, its frequent use clearly contributed to the commendably low operative mortality rate (5.6 per cent overall) and transfusion requirement (mean 5.5 units blood) in a series that included 25 extended right hepatectomies (5 deaths in this group). However, the need to cross-clamp the aorta as a preliminary step in 7 patients is not explained. There were only two deaths from uncontrolled haemorrhage, one actually on the operating table; thus venous bypass seems seldom likely to be required.

Several other lessons emerge from this important study.

1. The duration of ischaemia had no adverse effect on either liver function, morbidity, mortality or length of hospital stay, even though vascular inflow control was prolonged beyond 45 minutes in 23 patients (for up to 70 minutes); no attempt was made to cool the ischaemic liver, but body temperature fell spontaneously during the period of cross-clamping. Huguet has previously demonstrated the tolerance of the human liver to normothermic ischaemia[3]. These data cast doubt on the need to release the clamp at the porta hepatis every 15 minutes, although this is our current practice.
2. Cirrhosis undoubtedly increases the risks of liver resection, yet it may still be safely accomplished provided that major bleeding is avoided. There were two deaths in the cirrhotic group of 15 patients (13 per cent mortality), both following segmental resection, and postoperative liver failure was commoner than in non-cirrhotics (33 versus 3 per cent).
3. Most of the serious complications of hepatectomy are the consequence of haemorrhage either directly or indirectly. Subphrenic abscess and pleural effusion probably result from an undrained haematoma.
4. Some of the blood loss associated with difficult re-operations accompanies preliminary mobilisation of the liver and freeing of adhesions and will not be affected by vascular exclusion. Eleven patients lost an appreciable amount of blood before the liver itself was incised.
5. Blood transfusion makes an independent contribution to multiple organ failure, probably by aggravating coagulopathy: all the more reason, therefore, to avoid haemorrhage.

I think everybody would accept the authors' cogent argument that since blood loss at operation is the dominant factor affecting early outcome in major liver surgery, "the first operative priority should be to reduce bleeding". Opinions may differ on the best way of achieving this goal, but the present data clearly support the use of hepatic vascular occlusion in selected cases.

R.C.N. Williamson
Department of Surgery,
Royal Postgraduate Medical School,
Hammersmith Hospital, DuCane Road,
London W12 0NN, U.K.

### REFERENCES

1. Blumgart, L. H. (1988) Liver resection — liver and biliary tumors. In Surgery of the Liver and Biliary Tract, edited by L.H. Blumgart, vol. 2, pp. 1251–1280. Edinburgh: Churchill Livingstone
2. Bismuth, H. (1982) Surgical anatomy and anatomical surgery of the liver. *World Journal of Surgery*, **6**, 3–9
3. Huguet, C., Nordlinger, B., Bloch, P. and Conrad, J. (1978) Tolerance of human liver to prolonged normothermic ischaemia. *Archives of Surgery*, **113**, 1448–1451

### CAN PANCREATIC PHLEGMON BE DIAGNOSED?

### ABSTRACT

*Fan, S-t., Choi, T-k., Chan, F-l., Lai, E.C.S. and Wong, J. (1989) Pancreatic Phlegmon: What Is It? The American Journal of Surgery,* **157** 544–547

**In a retrospective study of 264 patients with acute pancreatitis, 22 were identified as having phlegmon by combined radiologic and clinical criteria. The radiologic criteria consisted of demonstration of abnormal lesion on computed tomography scan which was composed of masses of mixed density, free of extraluminal gas and lacking a well-defined wall. The clinical criteria was that the clinical course was free of sepsis. Half of the group thus identified had severe pancreatitis as defined as having three or more poor prognostic signs. Fever, leukocytosis, and serum amylase elevation persisted for a longer period than usual. Complication was infrequent but the lesion could persist for 3 to 4 months without producing symptoms. This is a relatively benign condition and surgery should be avoided**

### PAPER DISCUSSION

**KEYWORDS**: Pancreatitis, Pancreatic phlegmon

The term phlegmon seems to excite great interest among those attempting to describe acute pancreatitis and to understand its variegated clinical course. In large part that interest has been in trying to prove that the term is either bad or unuseful. There clearly has been no agreement on what the term means. Its original proponents used it primarily to denote a sterile process, with varying amounts of necrosis, but they also recognized that occult infection could be present or that infection could develop at a later time in the evolution of the disease.[1,2]

In the study by Fan *et al.*, the authors describe 22 patients who met their criteria of an abnormal inflammatory mass involving the pancreas and varying amounts of peripancreatic tissues, but without evidence of either pseudocyst or infection. Necrosis of the involved tissues was present in some of the cases and was associated with multi-system organ failure in four patients and death in three of those.

